# MOBILITY-RELATED PHYSICAL FUNCTION AND AGING IN PLACE FOR OLDER ADULTS WITH CHRONIC CONDITIONS

**DOI:** 10.1093/geroni/igae098.2952

**Published:** 2024-12-31

**Authors:** Marielle Jensen-Battaglia, Ying Wang, Robert Block, Kah Poh Loh, Supriya Mohile, Emily Agree, Christopher Seplaki

**Affiliations:** University of Rochester School of Medicine and Dentistry, Rochester, New York, United States; University of Rochester School of Medicine and Dentistry, Rochester, New York, United States; University of Rochester School of Medicine and Dentistry, Rochester, New York, United States; University of Rochester Medical Center, Rochester, New York, United States; University of Rochester Medical Center, Rochester, New York, United States; Johns Hopkins University, Baltimore, Maryland, United States; University of Rochester School of Medicine and Dentistry, Rochester, New York, United States

## Abstract

Chronic conditions may influence difficulty mobilizing inside the home (mobility), which presents a barrier to the goal of aging in place for many older adults. Understanding this relationship is critical as the prevalence of chronic conditions increases. In prior work (currently under review) we used data from community-dwelling adults aged ≥65 participating in ≥2 consecutive rounds (2011-2019) of the NHATS to show self-reported mobility difficulty was positively associated with moves to homes with fewer environmental barriers or more household members, but not to other destinations (e.g., homes with more barriers, nursing home/residential care). In the current analysis we use this dataset of 25,490 person-years of follow up from 5,894 unique participants who moved 1,456 times to investigate modification of this association by chronic conditions [non-skin cancer (5,472/25,490 person-years), myocardial infarction/stroke (6,621/25,490 person-years), dementia (8,011/25,490 person-years)]. All chronic conditions were positively associated with relocation to nursing home/residential care in bivariate multinomial logistic regression models (cancer: OR 1.43; 95% CI 1.02, 2.02; myocardial infarction/stroke OR 1.43; 95% CI 1.04, 1.96; dementia OR 2.87; 95% CI 2.08, 3.96). After adjusting for confounders, only dementia remained associated with moves to nursing home/residential care (OR 1.40; 95% CI 1.06, 1.85) for those without any mobility difficulty. However, the association of mobility with relocation did not vary significantly by chronic condition (p for interaction >0.05 for all conditions and relocation destinations). These results highlight mobility difficulty and barriers in the home environment as potentially modifiable factors to promote aging in place, regardless of chronic conditions.

